# Transfer of Care Between the Emergency Department and an Older People’s Mental Health Inpatient Unit

**DOI:** 10.1192/bjo.2025.10584

**Published:** 2025-06-20

**Authors:** Rachael Elliott

**Affiliations:** Sheffield Teaching Hospitals NHS Foundation Trust, Sheffield, United Kingdom. University of Liverpool, Liverpool, United Kingdom

## Abstract

**Aims:** Two patients within an Older People’s Mental Health (OPMH) unit that had been transferred to the emergency department (ED) for physical health assessment were found to have returned without a handover or discharge letter. The discharge letters, received more than 48 hours later, revealed important interventions that were consequently delayed. This audit aimed to identify compliance with NICE Quality Standard QS174: adults admitted with a medical emergency and whose care is being transferred to a different healthcare setting have information about their condition and needs passed onto their new care provider.

**Methods:** A retrospective audit using online records from 30 patients admitted to a Doncaster OPMH unit between January–June 2024. Records were reviewed for key terms to identify patients with ED visits and then assessed for (a) handover documentation, (b) discharge letter availability, (c) actions required for the psychiatry ward, and (d) the nature and implementation of these actions.

**Results:** There were 20 individual ED reviews. 35% (n=7) had a documented handover to the OPMH unit at point of return (POR). 65% (n=13) had no evidence of a handover. Discharge letters were available on average 4 days (range 1–14 days) post-discharge. 5 reviews had no handover documentation or letter in the records and were followed up separately to this audit.

8 reviews resulted in actions for the ward. 7 required medication changes (antibiotics, anticoagulants, diuretics, beta blockers), 1 required patient isolation. Only one patient had evidence of a handover and subsequently their new medication was started without delay. The remaining patients received medication no longer indicated or missed doses of new medication. Patient isolation occurred with significant delay. Two reviews had a discharge letter at POR, the remaining had discharge letters available 2–8 days post-discharge.

**Conclusion:** The handover process between the ED and OPMH unit requires formalization. Current ambiguity has led to reliance on delayed discharge letters for follow up.

Verbal handovers may be happening but there remains an unexplained delay in actions until the discharge letter is received. There is also evidence that discharge letters have been received and not escalated. Further discussions are required to determine whether the primary issue lies with an absence of handovers or letters from the ED or internal communication and escalation within the OPMH unit.

Collaborative work to improve patient handovers is being undertaken with the ED and awareness has been raised within the trust to ensure proactive effort to obtain and escalate handovers.

